# Identification and verification of the ferroptosis- and pyroptosis-associated prognostic signature for low-grade glioma

**DOI:** 10.17305/bjbms.2021.6888

**Published:** 2022-03-09

**Authors:** Jie Wang, Jie Ren, Jifeng Liu, Linyun Zhang, Qihang Yuan, Bin Dong

**Affiliations:** 1 Department of Neurosurgery, First Affiliated Hospital of Dalian Medical University, Dalian, Liaoning, China; 2 Department of Oncology, First Affiliated Hospital of Dalian Medical University, Dalian, Liaoning, China; 3 Department of General Surgery, First Affiliated Hospital of Dalian Medical University, Dalian, Liaoning, China

**Keywords:** Ferroptosis, pyroptosis, prognostic signature, immunotherapy, low-grade glioma

## Abstract

Accumulating evidence reveals that ferroptosis and pyroptosis play pivotal roles in tumorigenesis of low-grade glioma (LGG). In this research, we aimed to classify molecular subtypes and further identify and verify a novel multigene signature in LGG on the basis of ferroptosis- and pyroptosis-related genes (FPRGs). Raw sequencing data and corresponding clinical data of LGG samples retrieved from The Cancer Genome Atlas and Chinese Glioma Genome Atlas databases were obtained for the training and validation datasets. Non-negative matrix factorization (NMF) clustering defined by FPRGs associated with prognosis was performed to classify molecular subtypes of LGG patients. Least absolute shrinkage and selection operator-support vector machine-random forest analysis was carried out to develop a FPRG signature to predict the survival and benefit of immunotherapy of LGG patients. NMF clustering defined by FPRGs with prognostic values acted to categorize LGG patients into two molecular subtypes with different prognosis, clinical traits, and immune microenvironments. A six-FPRG prognostic signature was constructed, accompanied by the optimal p-value. The AUC values of our signature exhibited great prognostic performances. Our signature was superior to other four well-recognized signatures in predicting the survival probability of LGG patients. Immune characteristics, tumor mutation profile, tumor stemness indices, MGMT methylation, and immunotherapy response biomarkers showed significant differences between high- and low-risk populations. Finally, a nomogram was created for quantitative prediction of the survival probability of LGG patients, with the AUC values of the nomogram being 0.916, 0.888, and 0.836 for 1-, 3-, and 5-year survival, sequentially. Overall, the FPRG signature may function as an effective indicator for the prognosis prediction and immunotherapy response of LGG patients.

## INTRODUCTION

As tumors develop in the central nervous system, gliomas are categorized by the World Health Organization (WHO) into four grades based on their histopathological characteristics, with the WHO Grade II and III gliomas being regarded as low-grade gliomas (LGG) [1]. Apart from a long-term history of ionizing radiation, the risk factors leading to LGG are not thoroughly comprehended [2]. LGG proliferates and progresses in a variety of ways, and survival status is not satisfactory [3,4]. Patients with the WHO II and III gliomas have a median overall survival period of 78.1 months and 37.6 months, respectively [5].

Notwithstanding the recent advancements in diagnostic and treatment procedures, LGG can deteriorate into high-grade glioma in certain individuals, resulting in decreased treatment responses and a worse prognosis. It has been generally accepted that immunotherapy acts to improve the prognosis of certain patients with malignant tumors; however, identifying the people who benefit from immunotherapy is a critical yet challenging task. Therefore, developing and validating new prognostic signatures to better predict the clinical outcomes and immunotherapy of patients with LGG are still urgently required.

Cell death, consisting of apoptosis, necrosis, ferroptosis, parthanatos, oxeiptosis, oncosis, pyroptosis, and autophagy, exerts a considerable function in the pathogenesis and progression of cancer [6,7]. Of note, ferroptosis and pyroptosis have become a hot topic in recent years. Ferroptosis, named by Dr. Brent R. Stockwell in 2012, is an iron-dependent cell regulatory death mode caused by the accumulation of lipid peroxidation products and reactive oxygen species [8]. Researchers have found that the proliferation of glioma cells can be suppressed through activating ferroptosis [9]. In 2021, Wan et al. and Zheng et al. revealed that ferroptosis served as a new prognostic biomarker of LGG patients [10,11]. Besides, pyroptosis has been known as a pro-inflammatory type of modulated cell death that relies on the enzymatic activity of inflammatory proteases belonging to the cysteine-dependent aspartate-specific proteases family (caspases) [12].

The modulation of pyroptosis intermediated by caspase 1 in glioma cells might inhibit cell proliferation and migration, suggesting a potential new therapy for glioma interventions. However, as of today, the prognostic performances of pyroptosis in LGG have not been clearly discussed and the comprehensive effect of ferroptosis and pyroptosis in LGG has not yet been evaluated systematically. For this reason, more insight into ferroptosis and pyroptosis in LGG is still required to provide an in-depth comprehension.

In this research, we examined ferroptosis- and pyroptosis-related genes (FPRGs). We utilized DNA methylation, gene expression levels, and clinical data from the Chinese Glioma Genome Atlas (CGGA) and The Cancer Genome Atlas (TCGA) databases to conduct a complete bioinformatics analysis. After identifying FPRGs with prognostic values, we conducted non-negative matrix factorization (NMF) clustering to categorize LGG patients into completely different molecular subtypes with significantly different prognoses, clinical traits, and immune microenvironments.

Machine learning further filters the optimal model based on the FPRGs with prognostic values. Subsequently, we constructed and validated a risk score system of LGG with the optimal prognostic performance based on the TCGA and CGGA cohorts. In addition, immune cell infiltration, immune checkpoint gene expression, immune subtype identification, tumor mutation profile, tumor stemness indices, O6-methylguanine DNA methyltransferase (MGMT) methylation, and immunotherapy response biomarkers were analyzed between the low- and high-risk cohorts to examine the possible mechanisms and pathways associated with FPRGs, laying the groundwork for determining the beneficiaries of immunotherapy. Our findings illustrated that a novel FPRG signature may serve as an effective marker for the survival and immunotherapy response in patients with LGG.

## MATERIALS AND METHODS

### Data collection and processing

The fragments per kilobase of transcript per million mapped read values for LGG RNA-Seq data were acquired from TCGA, which served as the derivation cohort (529 LGG samples, 56,753 genes). In addition, mRNA data and matched clinical data on LGG were retrieved from the CGGA database, which served as the validation cohort (625 samples, 23,271 genes). For further analysis, all data retrieved from the CGGA and TCGA repositories were transformed into log2(x+1) form.

To assure that the genes used for constructing the panel are the shared genes in both derivation and validation cohorts, the “intersect” function in R was applied to intersect all genes from the two cohorts, and 17,818 genes were preserved. The “ComBat” function in the “sva” R package was utilized to batch normalize the data on gene expression profiles from the two cohorts.

Thirty-three pyroptosis-related genes and 259 ferroptosis-related genes were identified from the literature. After deleting the two ferroptosis and pyroptosis shared genes (GPX4 and IL6), we actually acquired 290 well-recognized FPRGs. Subsequently, we combined these 290 genes with their corresponding expression profiles in TCGA and CGGA cohorts, and only 260 genes were preserved with complete expression values for further analysis (TCGA: Supplementary Table S1 and CGGA: Supplementary Table S2).

**TABLE 1:**
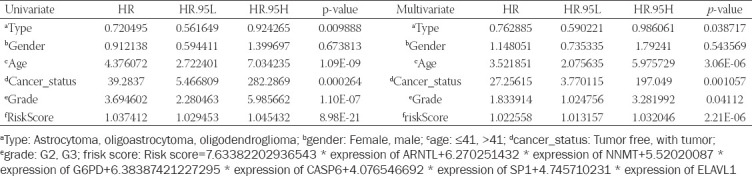
Univariate and multivariate Cox regression analysis determined the independent prognostic performance of our risk score

### NMF clustering identification of molecular subtypes

To decrease the dimensions of NMF clustering, univariate Cox regression analysis was applied to determine the genes that had prognostic values in both derivation and validation cohorts. Only prognosis-related genes closely related to ferroptosis and pyroptosis were preserved to serve as the dimensions and parameters of NMF clustering.

The “NMF” package in R was applied to cluster the LGG samples predicated on the expression profile of FPRGs with prognostic values, with the adjusted number of clusters as 2–10. The standard “brunet” option was selected, and 100 iterations were performed. The most appropriate clustering number was determined based on the NMF rank surveys and discrimination between different cluster subtypes.

### Comparison of the clinical outcomes, clinical traits, and tumor immune microenvironment between different molecular subtypes

Kaplan–Meier analyses including progression-free survival (PFS) and overall survival (OS) were applied to evaluate the prognostic performances of clusters. The fisher test was employed to the compositional differences of clinical traits between different subtypes. The “estimate” package in R was employed to compute the ImmuneScore, StromalScore, EstimateScore, and tumor purity of each LGG sample, and the “ggpubr” package in R was used to visualize this result. The CIBERSORT algorithm was utilized to analyze the infiltration composition of 22 immune cells in each LGG sample. The “wilcox.test” function in R was then implemented to investigate the discrepancy in the immune cell infiltration and common immune checkpoint genes (ICGs, Supplementary Table S3) expression between different subtypes, and only results with statistical differences were displayed. Subsequently, we intensively investigated the association between these immune checkpoints and clinical outcomes of LGG patients.

### Identification of the risk signature with the optimal multigene combination based on machine learning

To eliminate collinearity and classification error, the “glmnet” and “e1071” packages in R were utilized to carry out the least absolute shrinkage and selection operator (LASSO) and support vector machine (SVM) analysis of FPRGs with prognostic values, respectively. Subsequently, the “randomForestSRC” package in R was employed to conduct random survival forest analysis to determine the optimal prognostic signature combination with the aid of the risk scores of each LGG patient (risk score = ∑_k=1_^n^ expk*βk, βk is the gene coefficient of previous univariate Cox regression analysis). The best gene combination or the final signature was screened utilizing the log-rank p values by Kaplan–Meier (KM) analysis.

The samples were then stratified into low- and high-risk LGG populations according to the median risk scores in the derivation cohort. Survival analysis including OS and PFS by the Kaplan–Meier method was performed to estimate the prognostic performance of the prognostic signature in the derivation cohort. Receiver operating characteristic (ROC) curves were charted to validate the diagnostic values anticipating 1-, 3-, and 5-year survival rates based on the survival ROC package in R. t-distributed stochastic neighbor embedding (t-SNE) and principal component analysis (PCA) were performed to explore the distribution of different subgroups.

Furthermore, the predictive ability of our FPRG prognostic signature was compared with other four well-recognized prognostic signatures (an autophagy-related prognostic signature developed by Guo et al., an immune-related prognostic signature developed by Zhang et al., a RNA methylation-related prognostic signature constructed by Zheng et al., a ferroptosis-related prognostic signature developed by Liu et al.). All of the genes needed to create Guo’s, Zhang’s, Zheng’s, and Liu’s prognostic signatures were acquired. For further analysis, the gene expression profiles, as well as the matched clinical data, were preserved. Then, the “limma,” “survival,” “survminer,” and “timeROC” packages in R were utilized to estimate the predictive performance of our FPRG signature, Zheng’s signature, Guo’s signature, Liu’s signature, and Zhang’s signature. Subsequently, the “survival,” “survcomp,” “ggplot2,” and “ggpubr” packages in R were utilized to calculate the C-indices of the above signatures.

### Clinical characteristics, immune characteristics, and tumor stem characteristics in low- and high-risk populations

To examine the relationship between the FPRG prognostic signature and clinicopathological traits, fisher tests were applied to show the distribution differences of histological type, gender, survival status, age, cancer status, and grade between the low- and high-risk populations. The “ESTIMATE” R package was utilized to assess the discrepancy in the immune characteristics between low- and high-risk populations (predicated on the StromalScore, ImmuneScore, EstimateScore, and tumor purity) utilizing the transcriptome data. At the same time, on the basis of the FPRG signature, the XCELL, CIBERSORT, MCPCOUNTER, QUANTISEQ, CIBERSORT-ABS, and TIMER algorithms were compared to evaluate cell immune responses or cellular components between low- and high-risk populations.

A heatmap was used to detect changes in immune response with varied algorithms. The discrepancy in the ICGs expression levels between low- and high-risk populations was also explored. Only the immune cells and ICGs with significant statistical differences were displayed. In 2018, Thorsson’s article published in the “Immunity” journal [13] detected six immune subtypes (wound healing (C1), IFN-γ dominant (C2), inflammatory (C3), lymphocyte depleted (C4), immunologically quiet (C5), and TGF-b dominant (C6)) for more than 10,000 tumors across 33 cancer types of TCGA. To comprehensively explore the different immune features, we then investigated the discrepancy in immune subtypes between low- and high-risk populations with the aid of the findings provided by Thorsson et al.

In 2018, Melta’s article published in the “Cell” journal [14] applied one-class logistic regression (OCLR) machine learning method and extracted epigenetic and transcriptomic feature sets from non-transformed pluripotent stem cells as well as their differentiated progeny. OCLR-based epigenetic and transcriptomic signatures were utilized with regard to all pan-cancer 33 TCGA cohorts to calculate the DNA stemness scores (DNAss) and RNA stemness scores (RNAss). We also intensively explored the correlation between DNAss/RNAss, and risk score calculated by FPRG signature.

### Tumor mutation profile, immunotherapy response prediction, and MGMT methylation in low- and high-risk populations

The mutation profile of each LGG sample was acquired from TCGA platform and the number of non-synonymous mutations was counted utilizing the “perl” language. The aggregate number of somatic gene coding errors, deletion errors, gene insertion errors, and base substitutions identified per million bases was described as the tumor mutation burden (TMB). The differences in TMB between the low- and high-risk populations were computed, and the criteria for statistical significance were designated as *p* < 0.05. The association between TMB and the risk score computed by our FPRG signature was investigated utilizing the Spearman correlation coefficient. The LGG driver genes were then identified using the R package “maftools,” and the state of the topmost 20 genes with the highest frequency of mutation in the low- and high-risk populations was investigated further. We also explored the discrepancy in the clinical outcomes between different TMB scores and FPRG risk scores through K-M log-rank test.

Although TMB appears to be a reliable immune checkpoint blockade (ICB) response biomarker, its computation across multiple groups and platforms remains uncertain for the tumor mutation assessment results could be intervened by various sample types, experimental platforms, and computational mutation callers. Thus, we subsequently applied tumor immune dysfunction and exclusion (TIDE) database (http://tide.dfci.harvard.edu/) to predict immunotherapy response. TIDE, an online prediction tool, assists oncologists in predicting if a patient will respond to ICB treatment based on numerous biomarkers. These important biomarkers of immunotherapy consist of (1) IFNG average expression of interferon-gamma response signature, (2) CD274 gene expression value of PD-L1, (3) CD8 gene expression average of CD8A and CD8B, (4) exclusion and MDSC enrichment scores premised on the gene expression signatures of myeloid-derived suppressor cell and T-cell exclusion, and (5) T-cell-inflamed signature (Merck18). The “chisq.test” package in R was employed to compare the discrepancy in the composition of immunotherapy response between high- and low-risk populations. The “wilcox.test” platform in R was also utilized to compare the discrepancy in above immunotherapy response biomarkers between high- and low-risk populations.

It has been accepted that O6-methylguanine-DNA methyltransferase (MGMT) promoter methylation status and corresponding expression levels played a critical function in the development, chemotherapy, and prognosis of glioma patients. Thus, we intensively investigated the association of MGMT and FPRG signature. The raw DNA methylation data were downloaded from TCGA database through the “gdc-client.exe” software. MGMT gene expression and methylation status were extracted from transcriptomic data and methylation data, respectively. The “stat_compare_means” package in R was employed to compare the MGMT expression and methylation status between low- and high-risk populations. The “cor.test” function in R was applied to explore the correlation between MGMT expression, MGMT status, and FPRG risk score.

### Independent prognostic performance of the FPRG signature and nomogram plot establishment

Multivariate and univariate Cox regression analyses based on risk scores and the clinicopathological traits identified in TCGA cohort (i.e., histological type, gender, age, cancer status, and grade) were implemented to confirm if the FPRG signature can function as an independent prognostic index. Subsequently, independent prognostic indices were chosen to plot a nomogram utilizing the “rms” module in R. In addition, we plotted a calibration curve to evaluate the degree of fitting between the actual and survival probabilities estimated by the nomogram.

### Validation of the prognostic performance of our FPRG signature in the CGGA cohort

According to the calculation formula and median risk score provided in TCGA cohort, we computed the risk score of each LGG sample within the CGGA cohort and then categorized these samples into low- and high-risk subgroups. To verify the prognostic performance of our FPRG signature, ROC curve and KM survival analysis were carried out. The t-SNE and PCA analyses were also performed to display the distributions of LGG samples in high- and low-risk subgroups.

Likewise, fisher tests were applied to analyze the correlation between our FPRG signature and clinical traits in the CGGA cohort. Similar algorithms including XCELL, CIBERSORT, MCPCOUNTER, QUANTISEQ, CIBERSORT-ABS, and TIMER were also employed to explore the discrepancy in the immune features between low- and high-risk populations. Finally, the expressions of ICGs in different risk populations were investigated as previously described. All statistical analyses in this paper were performed based on the R and perl languages (*: *p* < 0.05, **: *p* < 0.01, and ***: *p* < 0.001).

## RESULTS

### Data acquisition and processing

**FIGURE 1:**
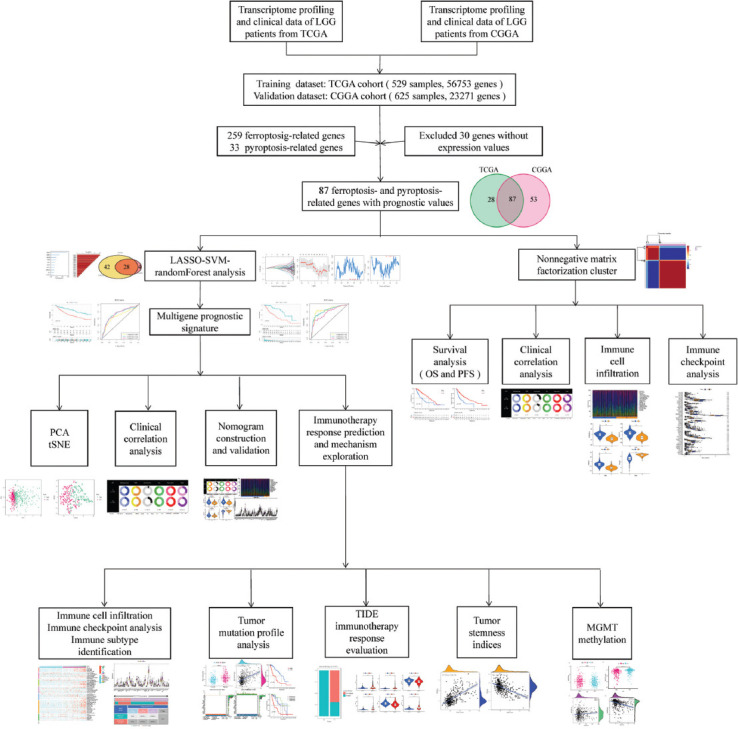
The workflow of the current study.

**FIGURE 2:**
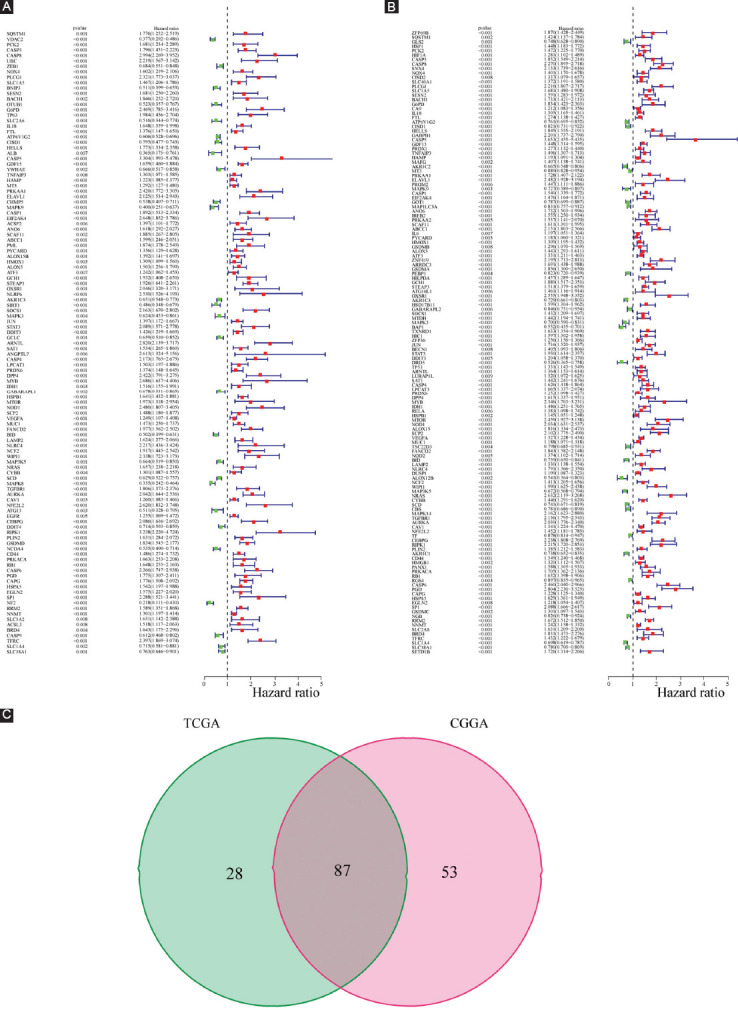
Identification of prognostic ferroptosis and pyroptosis-related genes (FPRGs). (A) One hundred and fifteen FPRGs with prognostic values in TCGA dataset; (B) 140FPRGs with prognostic values in the CGGA dataset; and (C) Venn diagram to identify 87 FPRGs with prognostic values in LGG.

Figure 1 depicts the flowchart for this research. LGG RNA-Seq data with clinical data were acquired from TCGA (529 LGG samples, 56,753 genes) as a training dataset and from CGGA (625 samples, 23,271 genes) as a validation dataset. Thirty-three pyroptosis-related genes and 259 ferroptosis-related genes were obtained based on the literature. Two hundred and ninety well-recognized FPRGs were obtained after deleting two ferroptosis and pyroptosis shared genes. Two hundred and sixty FPRGs with complete expression values both in the CGGA and TCGA cohorts were included in the following analysis. One hundred and fifteen FPRGs in the TCGA cohort and 140 FPRGs in the CGGA cohort were selected from the 260 FPRGs respectively through univariate cox regression analysis and false discovery rate adjustment (Figure 2A and B). Finally, 87 FPRGs with prognostic values were obtained after taking an intersection between the 115 prognostic FPRGs from TCGA cohort and the 140 prognostic FPRGs from the CGGA cohort (Figure 2C).

### NMF clustering identification of molecular typing based on the shared FPRGs with prognostic values

**FIGURE 3:**
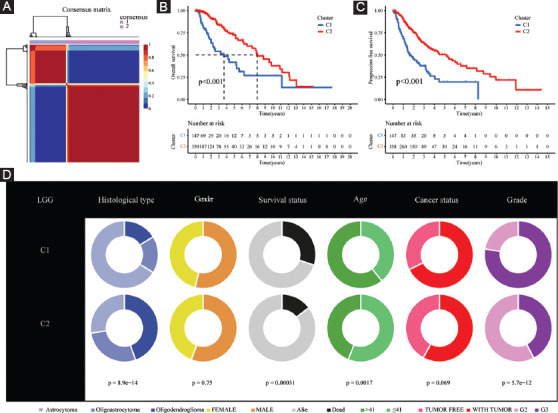
Non-negative matrix factorization clustering identification two molecular subtypes with significantly different prognosis and clinical characteristics. (A) The optimal clustering number of 2; (B and C) Kaplan–Meier analyses (OS and PFS) as regards two molecular subtypes; and (D) Pie charts illustrating the Chi-squared test of clinicopathologic factors between two molecular subtypes.

**FIGURE 4:**
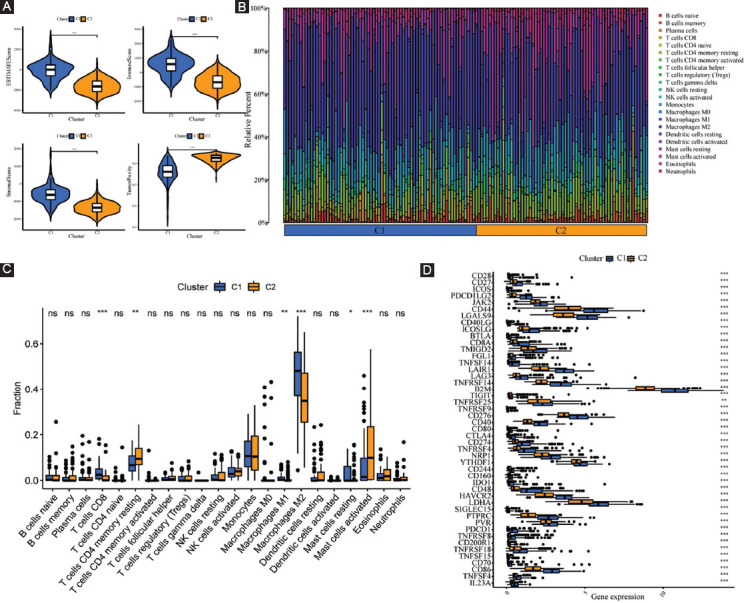
Systematic analysis of TME scores and immune cell infiltration in two molecular subtypes. (A) Comparison of TME components; (B) the proportions of 22 subsets of tumor-infiltrating immune cells in different subtypes; (C) discrepancy analysis of tumor-infiltrating immune cells in different subtypes; and (D) differential expression analysis of 47 immune checkpoints genes between two molecular subtypes.

**SUPPLEMENTARY FIGURE 1:**
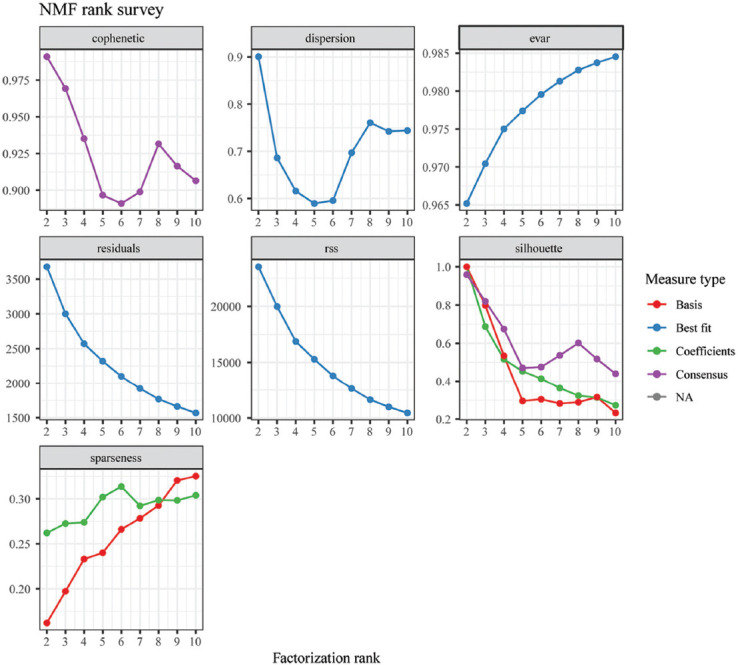
Rank survey of non-negative matrix factorization clustering.

**SUPPLEMENTARY FIGURE 2:**
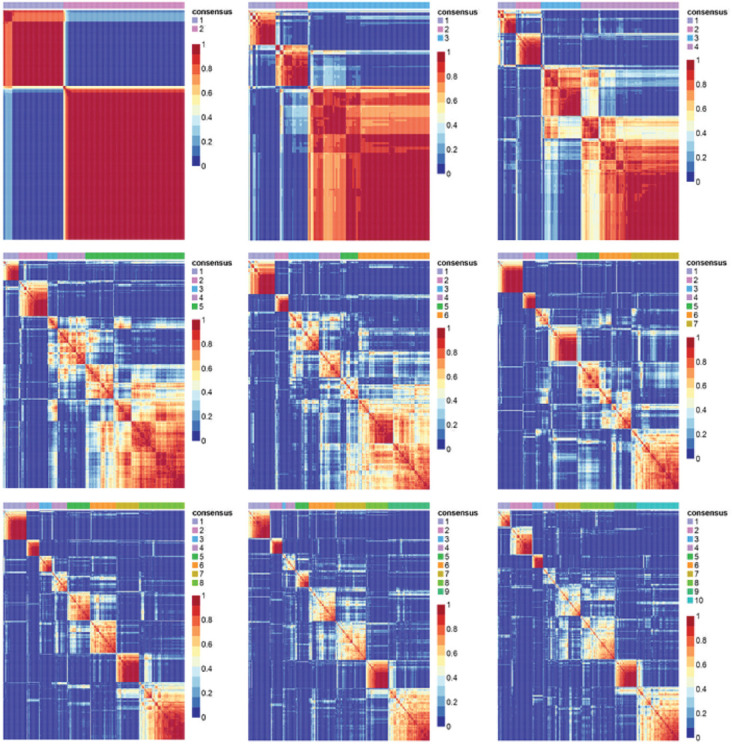
Consensus map of non-negative matrix factorization clustering.

The optimal clustering number of 2 is selected using the NMF algorithm on the basis of the cophenetic, dispersion, and silhouette indicators (Supplementary Figures 1 and 2, Figure 3A). The results of the following Kaplan–Meier analyses indicate that samples in cluster 2 (C2) are with better OS and PFS (Figure 3B and C). As shown in Figure 3D, the analysis about the compositional differences of clinical traits suggests that there are more astrocytoma samples in C1 and more oligodendroglioma samples in C2 (*p* = 8.9e-14). What’s more, there are more dead patients (*p* = 0.00031), old patients (>41 years old) (*p* = 0.0017), and patients with Grade 3 (*p* = 5.7e-12) in C1 compared with C2. In addition, TME components are estimated in C1 and C2, respectively, and the results in Figure 4A indicate that ImmuneScore, StromalScore, and EstimateScore are higher, but tumor purity is worse in C1. Of note, these scores have a positive correlation with the ratio of stromal components, immune components, as well as the total number of both components, implying that an increase in the scores contributed to an increase in the proportion of the matched components in the TME.

**FIGURE 5:**
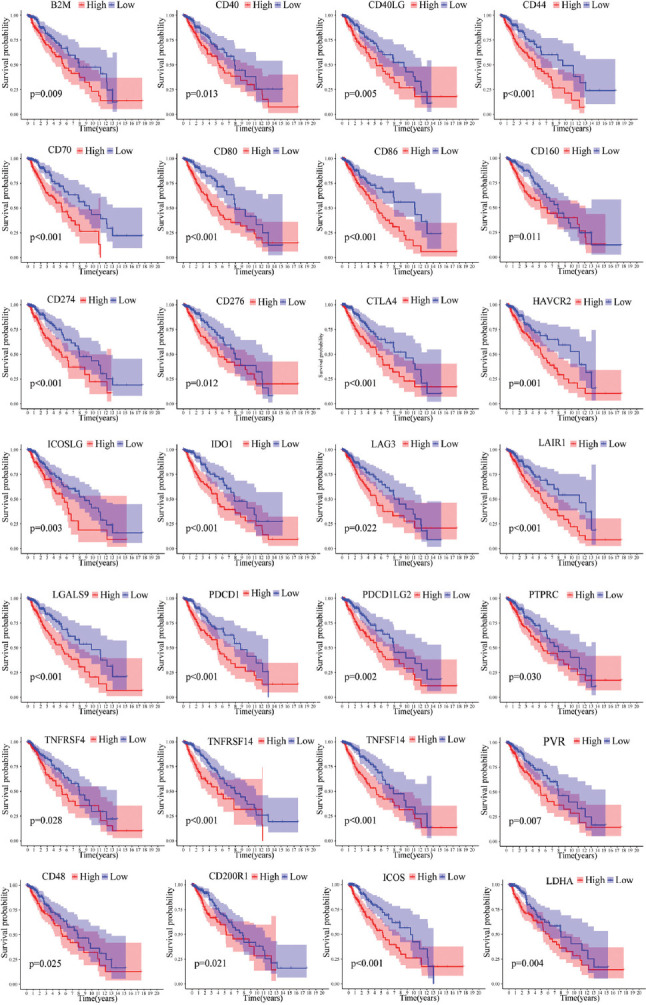
Kaplan–Meier analysis of 28 immune checkpoint genes. *B2M, CD40, CD40LG, CD44, CD48, CD70, CD80, CD86, CD160, CD200R1, CD274, CD276, PDCD1LG2, LGALS9, LAIR1, LAG3, HAVCR2, IDO1, CTLA4, ICOS, ICOSLG, LDHA, PDCD1, PTPRC, PVR, TNFRSF4, TNFSF14*, and *TNFRSF14* were prognostic relevant in LGG.

The CIBERSORT algorithmic technique was utilized to investigate the bulk gene expression profiles and thus deduce the percentages of 22 subsets of tumor-infiltrating immune cells in different subtypes (Figure 4B). C1 subtype features an increased infiltration of CD8+ T cells and macrophages; however, the C2 subtype is characterized by an increased infiltration of resting memory CD4+ T cells and activated mast cells (Figure 4C). Furthermore, the expression levels of all statistically different immune checkpoints genes are higher in C1 (Figure 4D). For further survival analysis about these involved immune checkpoints, low expression levels of the 28 immune checkpoints genes in total *(B2M, CD40, CD40LG, CD44, CD48, CD70, CD80, CD86, CD160, CD200R1, CD274, CD276, PDCD1LG2, LGALS9, LAIR1, CTLA4, LAG3, IDO1, HAVCR2, ICOS, ICOSLG, LDHA, PDCD1, PTPRC, PVR, TNFRSF4, TNFSF14, and TNFRSF14)* are connected with better survival probability (Figure 5).

### Identification and validation of FPRGs-based prognostic signature

**FIGURE 6:**
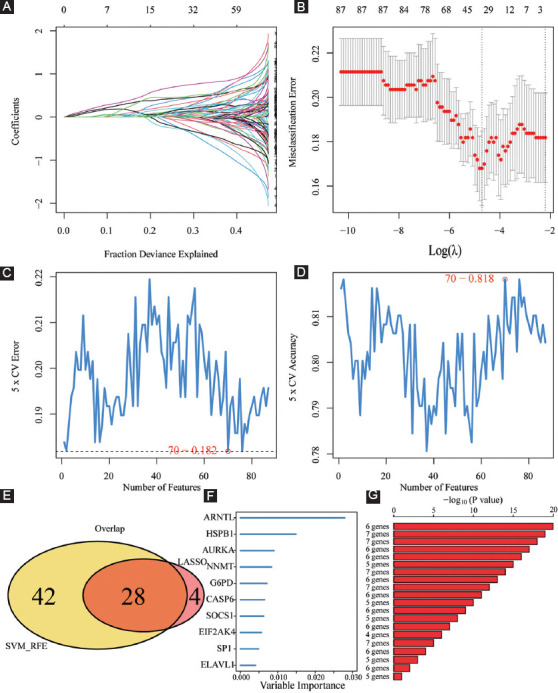
Machine learning identification of the optimal prognostic signature. (A and B) Identification of 32 FPRGs through the LASSO algorithm; (C and D) identification of 70 FPRGs through the SVM-RFE algorithm; (E) acquisition of 28 candidate FPRGs after intersecting LASSO and SVM-RFE algorithms; and (F and G) construction of a six-FPRG signature through random survival forests variable hunting algorithm.

**FIGURE 7:**
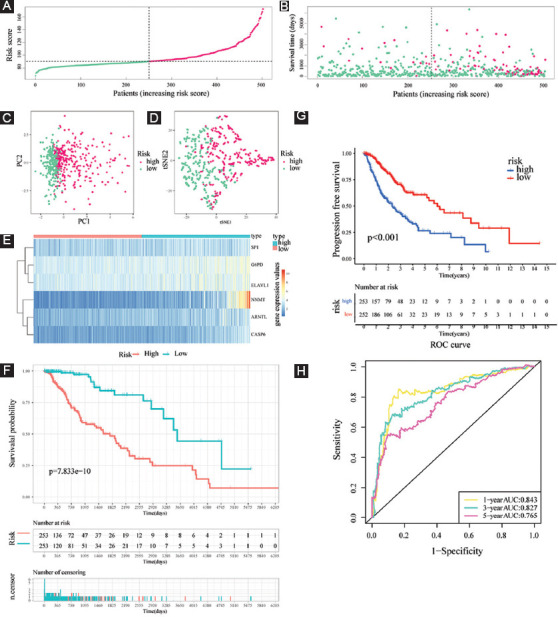
Evaluation of the prognostic value of risk score in the training cohort. (A and B) Distribution of risk score and patient survival time and status of LGG patients (A: the green and pink dots represent the low- and high-risk samples respectively; B: the green and pink dots represent the alive and dead samples respectively) ; (C and D) PCA and t-SNE analysis illustrated an excellent clustering performance of the six-gene-based risk score; (E) heatmap of the expression levels of six FERGs involved in the signature in the cohort; and (F and G) survival curve of training cohort; (H) ROC curves of training cohort.

After acquiring 87 prognostic FPRGs, we also performed the LASSO algorithm to obtain a set of 32 FPRGs (Figure 6A and B) and the SVM-RFE algorithm to choose a set of 70 FPRGs (Figure 6and D). Following the intersection of the FPRGs filtered out through the LASSO and SVM-RFE algorithms, 28 candidate FPRGs were found to perform random survival forests variable hunting algorithm to screen the genes further. Then, a novel FPRGs-based signature is identified because it has a relatively big (−log10) *p*-value and risk score can be calculated as follows: Risk score = 4.076546692 * expression of SP1+5.52020087 * expression of G6PD+4.745710231 * expression of ELAVL1+ 6.270251432 * expression of NNMT+7.63382202936543 * expression of ARNTL+6.38387421227295 * expression of CASP6 (Figure 6E-G). Subsequently, samples in training cohort are categorized into low- and high-risk subgroups according to the median risk score of 89.24308 (Figure 7A, Supplementary Table S4).

**SUPPLEMENTARY FIGURE 3:**
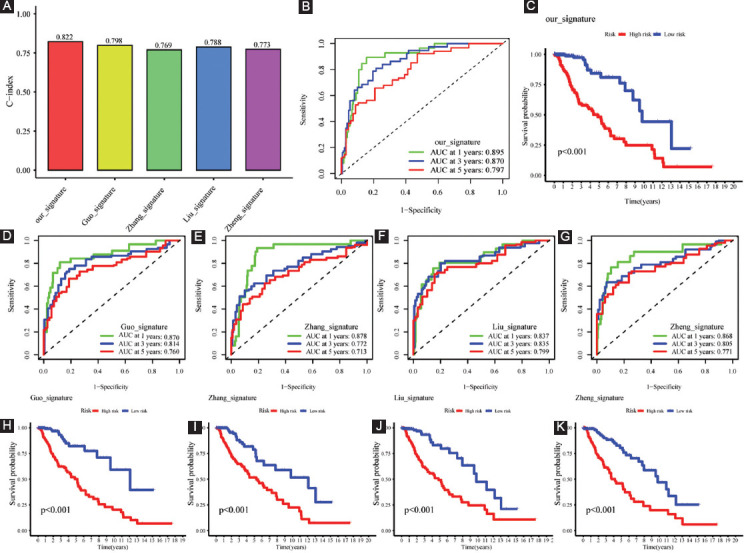
Comparative analysis. (A) The C-indices of our, Guo’s, Zhang’s, Liu’s, and Zheng’s signatures; (B-C) ROC curves and survival analysis of our signature; (D-G) ROC curves of Guo’s, Zhang’s, Liu’s, and Zheng’s signatures; and (H-K) survival analysis of Guo’s, Zhang’s, Liu’s, and Zheng’s signatures.

The survival status and risk scores distributions suggest that patients with high-risk scores were more likely to die (Figure 7B). t-SNE and PCA were then utilized to determine the overall distribution of LGG patients in low- and high-risk populations. The patients within the two groups can be effectively differentiated (Figure 7C and D). The levels of the six FPRGs expressions in our signature are shown by a heatmap in Figure 7E, which is harmonized with the values in the calculation equation (Figure 7E). The following survival analysis illustrated that patients with high-risk scores had worse OS and PFS (all *p* < 0.001) (Figure 7F-G). In addition, the ROC curves were utilized to verify the diagnostic values. The AUC values of the ROC curves are 0.843, 0.827, and 0.765 for 1-, 3-, and 5-year survival (Figure 7H). Notably, compared with another four well-recognized prognostic signatures, our signature shows a superior probability for survival prediction. The C-index of our signature is 0.822, while the C-indexes of Guo’s signature, Zhang’s signature, Liu’s signature, and Zheng’s signature are 0.798, 0.769, 0.788, and 0.773, respectively (Supplementary Figure 3).

### Clinical characteristics, immune characteristics, and tumor stem characteristics in low- and high-risk populations

**FIGURE 8:**
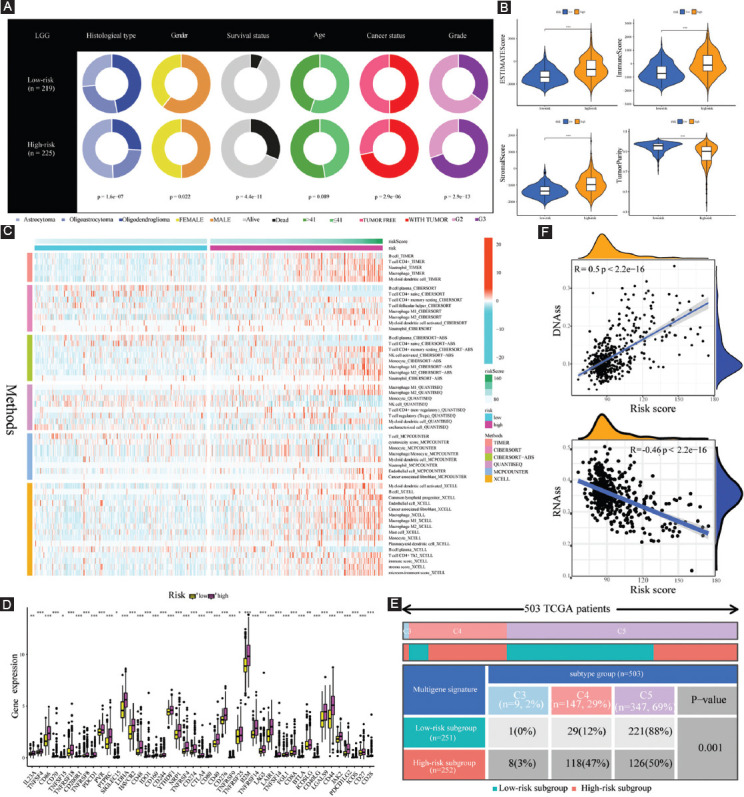
Clinical characteristics, immune characteristics, and tumor stem characteristics in the training cohort. (A) Pie charts illustrating the Chi-squared test of clinicopathologic features between low- and high-risk subgroups; (B) comparing TME components between low- and high-risk subgroups; (C) the landscape of the distribution of immune cell infiltration in the training cohort; (D) the expression levels of ICGs in the training cohort; (E) heatmap and table showing the distribution of immune subtypes (C3, C4, and C5) between low- and high-risk subgroups; and (F) the correlation analysis between tumor stemness index and risk score.

As illustrated in Figure 8A, the low-risk population possessed a higher proportion of oligodendroglioma samples and a lower proportion of astrocytoma samples, while the opposite was the case for the high-risk subgroup. In addition, there are more female, dead patients, with tumor patients, and G3 patients in the high-risk subgroup (all *p* < 0.05).

To investigate the specific function of FPRGs in TME, ESTIMATE algorithm was employed to assess the association of tumor purity, immune purity, stromal purity, and FPRG scores. Our results revealed that high-risk populations exhibited enhanced levels of ImmuneScore, StromalScore, and EstimateScore, but showed attenuated levels of tumor purity (Figure 8B).

To further explore the abundance of immunocyte infiltrating in the tumor microenvironment (TME), a variety of algorithms were applied to estimate the percentage of the immune cell infiltrate in high- and low-risk LGG populations. As depicted in Figure 8C, the high-risk population showed an enhanced proportion of B cells based on TIMER and XCELL algorithms; however, the proportion of plasma cells was considerably lower in the high-risk population than that in the low-risk population based on CIBERSORT, CIBERSORT-ABS, and XCELL algorithms. Although T cell shows a lower proportion collectively based on MCPCOUNTER, various types of T cell show a wide variety of expression patterns. CD4+ T cells have a higher proportion in the high-risk population-based on TIMER. Among them, naive CD4+ T cells have a lower proportion, and resting memory CD4+ T cells have a higher proportion based on CIBERSORT and CIBERSORT-ABS. T helper 2 (Th2) cells have a higher proportion in the high-risk subgroup based on XCELL. T-cell regulatory (Treg) has a higher proportion in the high-risk subgroup based on QUANTISEQ. Follicular helper T cell (Tfh) has a lower proportion in the high-risk subgroup based on CIBERSORT. As for macrophage, it shows a higher proportion in the high-risk population premised on TIMER and MCPCOUNTER. Notably, both M1 macrophage and M2 macrophage are more in the high-risk population compared with the low-risk population on the basis of CIBERSORT, CIBERSORT-ABS, QUANTISEQ, and XCELL. In addition, myeloid dendritic cells have a higher proportion in the high-risk subgroup based on TIMER, CIBERSORT, QUANTISEQ, MCPCOUNTER, and XCELL, and plasmacytoid dendritic cells also show a higher proportion based on XCELL.

Increased immune cell infiltration may be the compensatory result of a low local immune response. As shown in Figure 8D, high-risk LGG populations exhibited enhanced expression of ICGs (all *p* < 0.05, Wilcox.test). Higher expression of ICGs acted to attenuate efficient anti-cancer immune responses, thereby inducing the migration of immunocytes into the TME to enhance compensatory response. Subsequently, the discrepancy of immune subtypes in Figure 8E suggests that all the LGG patients in TCGA cohort are only connected with C3, C4, and C5 immune subtypes. Compared with the low-risk subgroup, high-risk LGG populations consist of a higher proportion of C3 and C4 immune subtypes and a lower proportion of C5 immune subtypes (p = 0.001, chisq.test).

Tumor stem cell score is not only correlated with immune infiltration and immune checkpoints but also reveals the occurrence pattern of intratumor heterogeneity. An in-depth examination of tumor stem cell score aids in strengthening our understanding of the tumor immune microenvironment and developing new targeting drugs associated with ICB therapy. Thus, the correlation analysis was performed to explore whether our FPRG signature was correlated with tumor stemness index, and the findings indicated that the risk score had a positive correlation with DNAss (R = 0.5, *p* < 2.2e−16) and negatively correlated with RNAss (R = –0.46, *p* < 2.2e−16) (Figure 8F).

### Tumor mutation profile, immunotherapy response prediction, and MGMT methylation in high- and low-risk populations

**FIGURE 9:**
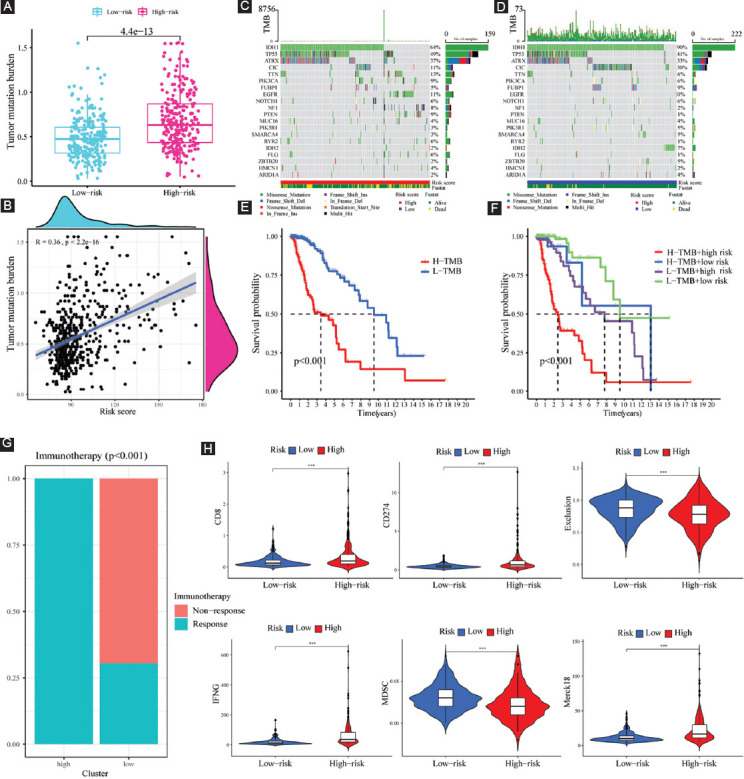
TMB analysis and immunotherapy response prediction in the training cohort. (A) TMB difference between high- and low-risk subgroups; (B) correlation analysis between risk score and mutation load; (C and D) OncoPrint of frequently mutated genes in high- and low-risk subgroups; (E) Kaplan–Meier curve of OS for patients represented by the samples classified by risk score; (F) Kaplan–Meier curve of OS for patients represented by the samples classified by both the risk score and TMB score; (G) the discrepancy of immunotherapy response in low- and high-risk subgroups; and (H) immunotherapy response prediction in the training cohort.

TMB was recently deemed as a novel prognostic biomarker that is closely associated with the response to immunotherapy. Considering its vital prognostic and clinical value, we evaluated the correlation between TMB and risk score, and the outcomes suggested that TMB was considerably elevated in the high-risk subgroup (*p* = 4.4e-13; Figure 9A). Moreover, Spearman correlation analysis illustrated that risk score has a moderately positive correlation with TMB (R = 0.36, *p* <2.2e-16; Figure 9B). Furthermore, we examined the changes in LGG driver genes across low- and high-risk subgroups. Driver genes with a high change frequency are shown in Figure 9C and D, such as IDH1, TP53, ATRX, CIC, and FUBP1. Of note, IDH1, CIC, and FUBP1 mutation frequency is higher in the low-risk subgroup; however, TP53 and ATRX mutation frequency is higher in the high-risk subgroup.

Patients with low TMB scores had a better chance of surviving than those with high TMB values (Figure 9E). To further distinguish the synergistic or antagonistic ability of the TMB and FPRG scores to predict survival, we classified the patients based on these two scores and carried out survival analysis. Those with low TMB and FPRG scores had the best prognoses, whereas patients with high TMB and FPRG scores had the worst prognoses (Figure 9F). These findings imply that the mutational burden may be linked to immunotherapy response, thereby providing a novel perspective on checkpoint blockade treatment.

As depicted in Figure 9G, considerable differences were found in the results of immunotherapy response between low- and high-risk populations. Specifically, high-risk LGG populations have higher scores of IFNG, CD274, CD8, and Merck18, and all of them are positive biomarkers for ICB therapy (Figure 9H). Besides, high-risk LGG populations have lower scores of T-cell exclusion signature and MDSC, which are negative biomarkers of ICB response (Figure 9H). Overall, the results of immunotherapy response prediction are consistent with those of TMB. That is to say, the FPRG score that we developed can effectively anticipate the response to immunotherapy and high-risk LGG patients have a higher likelihood of benefiting from ICB therapy.

**FIGURE 10:**
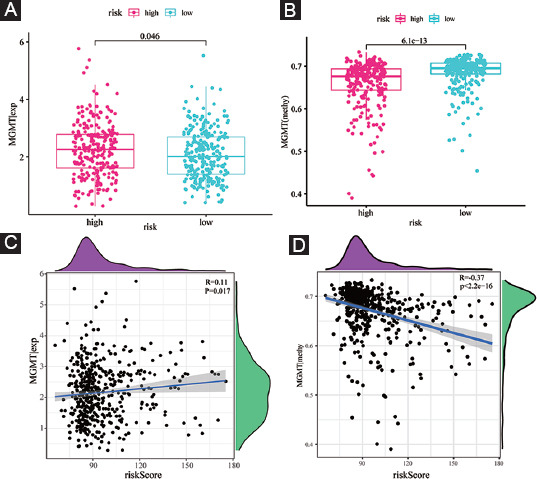
MGMT expression and methylation status in the training cohort. (A) The discrepancy of MGMT expression level between high- and low-risk subgroups; (B) the discrepancy of MGMT methylation status between high- and low-risk subgroups; (C) the correlation between MGMT expression level and risk score; and (D) the correlation between MGMT methylation status and risk score.

Growing numbers of studies uncovered that the methylation status of MGMT gene promoter is the key factor that determines the MGMT expression, and can influence the efficacy of chemotherapy as well as the prognosis of glioma patients. Thus, we further intensively investigated the potential association of MGMT and FPRG signature. Our results showed that compared with the low-risk subgroup, high-risk populations are accompanied by a higher MGMT gene expression and a lower MGMT methylation level (Figure 10A and B). We also found that there is a slightly positive correlation between risk score (R = 0.11, *p* = 0.017) and MGMT expression, and a moderately negative correlation between risk score and MGMT methylation (R = –0.37, *p* < 2.2e-16) (Figure 10C and D).

### Independent prognostic performance of our FPRG signature and nomogram plot establishment

**FIGURE 11:**
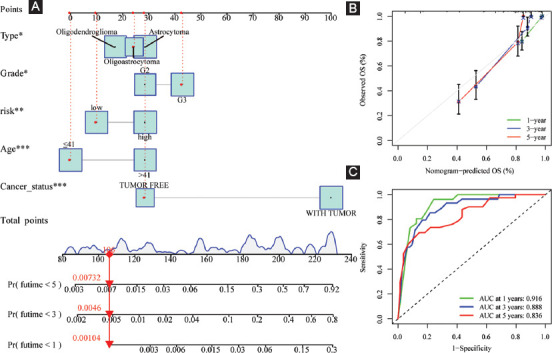
Creation and verification of the risk score-based nomogram plot. (A) A nomogram of LGG was used to predict 1-year, 3-year, and 5-year survival rates; (B) calibration curves for accuracy validation of the nomogram; and (C) the AUC values of the ROC curves for improved evaluation of the prognostic ability of the nomogram.

To identify whether our FPRG signature was an independent prognostic factor for OS, multivariate and univariate Cox regression analyses comprising tumor type, gender, age, cancer status, grade, and FPRG score were performed. The results showed that tumor type, age, cancer status, grade, and FPRG score could serve as an independent prognostic index (Table 1). Subsequently, a nomogram plot involving the above independent prognostic indicators was plotted for quantitatively predicting the survival probability of LGG patients (Figure 11A). Then, the predictive performance of the nomogram plot was validated utilizing calibration curves. The findings illustrated that the nomogram-predicted survival probabilities were consistent with the actual ones (Figure 11B). The AUC values of the ROC curves were 0.916, 0.888, and 0.836, respectively, which suggest a great prognostic performance of the nomogram (Figure 11C).

### Validation of the prognostic performance of our FPRG signature in CGGA cohort

**FIGURE 12:**
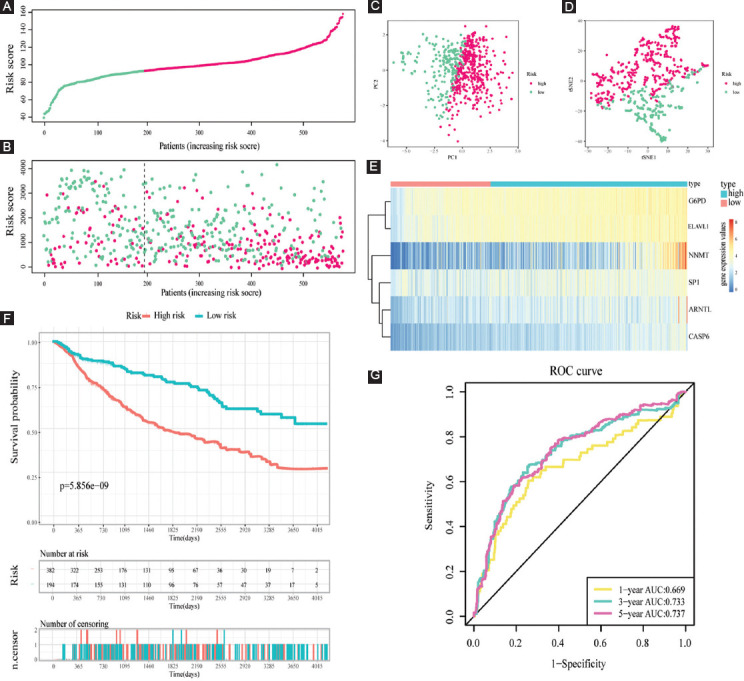
Validation of the prognostic value of risk score in the validation cohort. (A) Group division in the validation cohort (the green and pink dots represent the low- and high-risk samples respectively); (B) patients in the high-risk group had an increased incidence of death (the green and pink dots represent the alive and dead samples respectively); (C and D) PCA and t-SNE analysis demonstrated an excel-lent clustering performance of the six-gene-based risk score; (E) heatmap of the expression levels of six FPRGs involved in the signature in the validation cohort; (F) survival curve in CGGA cohort; and (G) ROC curves in CGGA cohort.

To highlight the robustness of our FPRG signature established from the TCGA cohort (i.e., training dataset), the patients from the CGGA cohort (i.e., validation dataset) were also categorized into low- and high-risk subgroups by the median value calculated with the same equation as that from the TCGA cohort (Figure 12A, Supplementary Table S5). On the whole, the results in the validation cohort are consistent with those in the training dataset. The patients with high-risk scores have a higher likelihood of dying and have a shorter survival time (Figure 12B). Similar to the results acquired from the training dataset, LGG patients can also be distinguished clearly through PCA and t-SNE analysis in the validation dataset (Figure 12C and D). The levels of the six FPRGs expressions in our signature are also consistent with the values in the calculation equation, as shown by the heatmap (Figure 12E). The findings from the survival analysis also suggested that patients in the high-risk subgroup exhibited poorer survival probability (Figure 12F). In the CGGA cohort, the AUC values of our FPRG signature were 0.669, 0.733, and 0.737 for 1-, 3-, and 5-year survival probability (Figure 12G).

**FIGURE 13:**
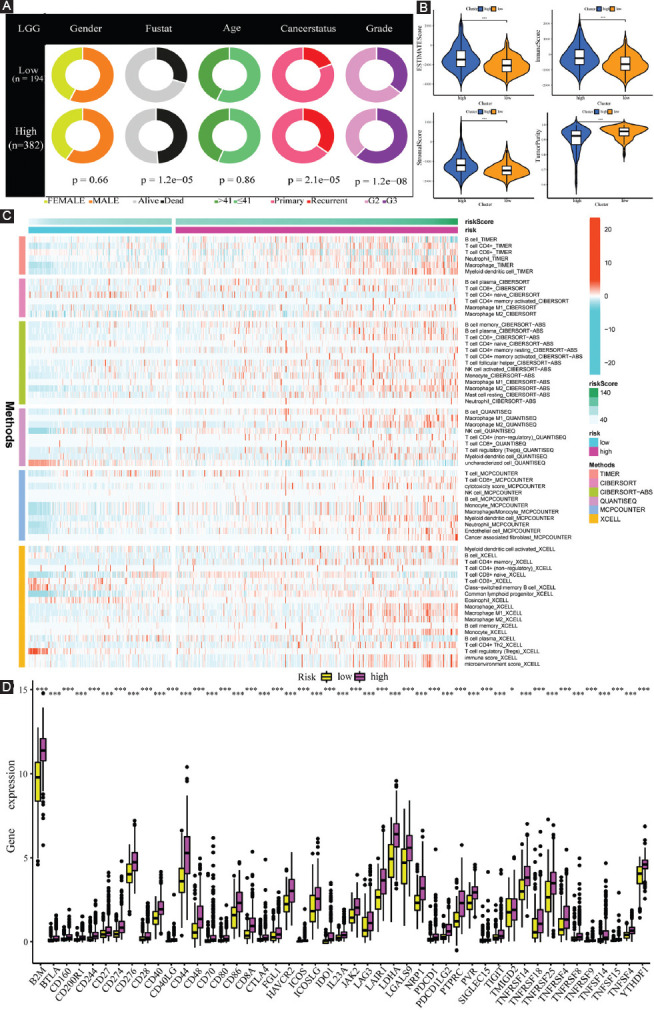
Clinical characteristics and immune characteristics in the validation cohort. (A) Compositional differences analysis of clinical traits between low- and high-risk subgroups; (B) comparison of TME components between low- and high-risk subgroups; (C) the landscape of the distribution of immune cell infiltration in the validation cohort; and (D) the expression levels of ICGs in the validation cohort.

Subsequently, the differences in the clinical characteristics between low- and high-risk populations are illustrated in Figure 13A. Thus, it is evident that the high-risk populations consist of more dead, more recurrent, and more G3 patients. These were just consistent with previous results that high-risk LGG patients were accompanied by worse prognoses. Likewise, high-risk populations in the CGGA cohort showed increased levels of EstimateScore, ImmuneScore, and StromalScore, and a decreased level of tumor purity (Figure 13B). The abundance of immunocyte infiltration in low- and high-risk populations is also depicted in Figure 13C. Similarly, high-risk populations showed an enhanced infiltration of B cells, CD4+ T cells, Th2 cells, Treg cells, macrophages, and myeloid dendritic cells. In addition to these, the discrepancy in the expression of ICGs between low- and high-risk populations demonstrated the same trends. Contrasted with low-risk populations, high-risk populations exhibited an enhanced expression of ICGs, which might be responsible for a likely compensatory increase in immune cell infiltration (Figure 13D).

## DISCUSSION

LGG has been identified as a group of primary brain tumors that progress from supporting glial cells. With unclear pathogenesis and unsatisfied therapeutic effects, in-depth mechanisms of LGG are supposed to be intensively investigated. Interestingly, ferroptosis and pyroptosis, as new forms of cell death, may have the potential to offer a novel strategy for the treatment of tumors. Hence, we aimed to classify molecular subtypes and further identify and verify a novel multigene signature to anticipate the prognosis and immunotherapy response of LGG patients based on FPRGs.

With sequencing data and corresponding clinical data of LGG samples from the TCGA as well as the CGGA databases, 87 FPRGs with prognostic values were identified for NMF clustering and signature construction. First, we obtained two molecular subtypes with significantly different prognoses, clinical traits, and immune microenvironment. The subtype with a worse prognosis is composed of more dead patients, old patients, and G3 patients. The TME is an intricate assembly of the tumor, immune, stromal, and extracellular components [15], so ImmuneScore, StromalScore, and EstimateScore were calculated to infer the stromal and immune components of each patient. The elevated StromalScore and ImmuneScore are associated with larger respective components in the TME. The results indicated that high-risk LGG patients may have greater immune abundance. A growing number of research reports have demonstrated that metabolic alterations may influence TME, particularly immune cells [16-18]. TME components are critical for the onset and progression of cancers. The targeting of TME remodeling might offer a prospective treatment approach to suppress tumor progression. Numerous research reports have shown that the immunological microenvironment has an impact on the biological activity of tumors [19-21]. Thus, the immune cells in different subtypes are displayed visually. The infiltration level of CD8+ T cells and macrophages was higher in the C1 subtype, whereas that of CD4+ T cells and activated mast cells was higher in the C2 subtype. Furthermore, the increased expression of a total of 28 immune checkpoint genes shows a relationship with a poor survival prognosis, which indicates that these populations may benefit from treatment with immune checkpoint inhibitors.

Then, a novel FPRG signature, involving specificity protein 1 (SP1), glucose-6-phosphate dehydrogenase (G6PD), nicotinamide N-methyltransferase (NNMT), ELAV-like RNA-binding protein 1 (ELAVL1), Aryl hydrocarbon receptor nuclear translocator like (ARNTL), and Caspase-6 (CASP6), was created and verified to anticipate the survival and benefit with immunotherapy, which contributes to the differentiation of patients into high- and low-risk subgroups in the training cohort and validation cohort, respectively. In the two cohorts, patients in the high-risk subgroup had a poorer prognosis, greater immune abundance resided in the high-risk subgroup and increased ICGs expression existed in the high-risk subgroup. It is noteworthy that our signature has a satisfactory diagnostic value and is superior to another four signatures for survival prediction.

There are a total of six genes included in the signature to be utilized for risk score calculating. The overexpression of transcription factor SP1 has been identified in diverse cancers, such as the brain (glioma), lung, pancreatic, and gastric [22-25]. In glioma, Sp1 is upregulated and enhances MMP-2-mediated cell invasion, which indicates a decreased survival [24]. G6PD performs a significant function in the synthesis of ribose and the reduction of equivalent nicotinamide adenine dinucleotide phosphate through the pentose phosphate pathway. Increasing the levels of G6PD mRNA expression levels facilitates the prediction of poor clinical outcomes in cancer patients, such as higher resistance to medication, migration, or proliferation of tumor cells [26]. ELAVL1 is one of the best-studied regulators of cytoplasmic mRNA fate [27]. Connected with the upregulation of LINC00336, it performs a vital function in lung cancer [28]. In addition, the pathogenesis of bladder cancer is promoted by recruiting ELAVL1 to stabilize target mRNAs [29]. What’s more, IGF2BP3/ELAVL1 complex leads to prolonged half-lives of cancer-related mRNA molecules and increased expression of the target genes [30].

The overexpression of NNMT has been found in diverse human cancers. NNMT is known to impair the methylation potential of cancer cells by exhausting methyl units from S-adenosyl methionine to form the stable metabolic product 1-methylnicotinamide [31]. As an independent risk factor, elevated stromal NNMT expression in CRC further suggests the dismal survival outcomes in patients in the initial stages of CRC (Stage I and II) as well as the patients undergoing chemotherapy [32]. ARNTL2, with an impact on metastatic capacity *in vivo* and clonal growth in cell culture, is capable of making lung adenocarcinoma metastatic self-sufficiency through an Arntl2-driven secretome [33]. It also plays a role in the survival prediction of pancreatic cancer [34]. The expression of ARNTL2 is elevated in a highly proliferative colon cancer cell line and in colorectal cancer and has been found to be associated with tumor aggressiveness and invasiveness [35]. Caspase-6 is a major modulator of host defense inflammasome activation and innate immunity [36]. Notably, Casp6 modulates the activation and the differentiation of B cells into plasma cells through the alteration of cell cycle entry [37].

The immune cells within TME perform a vital function in tumorigenesis. It is well-recognized that these tumor-related immune cells might have tumor-promoting or tumor-antagonizing roles. Research report has shown that although the tumor-antagonizing immune cells in TME seem to target and destroy cancer cells in the initial stages of tumorigenesis, these cancer cells ultimately evade immunological surveillance [38]. In subsequent in-depth exploration about the abundance of immunocyte infiltration, some cancer-promoting immune cells, including Th2 [39,40], Treg [41], M2 macrophage [42], and pDC [43], are upregulated in the high-risk subgroup, despite some anti-tumor immune cells are also had higher proportions, such as B cell [44], M1 macrophage [45], mDC [46,47], and Tfh [48,49]. To perform its function of anti-tumor, plasma cells showed lower proportions after large consumption in the high-risk subgroup. Simultaneously, naïve CD4+ T cells transfer to T-cell memory due to exposure to antigen [50]. Indeed, naïve CD4+ T cells decreased and T-cell memory increased in the high-risk subgroup. In addition, cancer cells have been found to possess the capacity of activating various immune checkpoint pathways that contain immunosuppressive functions [51]. Thus, cancer-promoting immune cells with statistically different expression levels and the ICGs with increasing expression levels in high-risk subgroup are expected to act as potential efficient therapeutic targets.

MGMT, also referred to as O6-methylguanine-DNA methyltransferase, is a DNA repair enzyme that assumes a crucial function in chemoresistance to alkylating agents, and the level of MGMT expression has a positive correlation with the degree of malignancy [52-54]. According to some researches, methylation of isolated regions of the CpG island of MGMT is associated with the silencing of the MGMT gene [55]. In this study, the upregulation of MGMT expression and downregulation of MGMT methylation in the high-risk subgroup indicate a poor prognosis.

TMB, together with PD-L1 expression, has been illustrated as a beneficial biomarker for the selection of ICB in several types of cancer [56]. In this study, an increase of TMB in the high-risk subgroup is also associated with an unsatisfied prognosis. Then, the top three genes (IDH1, TP53, and ATRX) with the high frequency of mutation were identified as driver genes of LGG. It is reported that LGG patients with IDH mutations and 1p/19q codeletions had the best prognosis [3]. TP53 mutations (94%) and ATRX inactivation (86%) were found in nearly all LGG patients with IDH mutations and no 1p/19q codeletion [3]. In addition, ATRX alteration strongly intersected with TP53 mutation (*p* < 0.0001) and IDH1/2 (*p* < 0.0001) in specimens in all WHO grades [57]. In the high-risk subgroup, higher mutation rates of IDH, TP53, and ATRX imply a potential correlation between the three genes and the collective alteration of these three genes might lead to developing a new therapeutic target. As a consequence, all these potential mechanisms that resulted in poor prognosis are supposed to apply to clinical treatment.

Significant statistical difference was observed for TIDE-derived immunotherapy response prediction between high- and low-risk LGG populations. Of note, our results uncovered that all patients in the high-risk subgroup might benefit from immunotherapy and only part of patients in the low-risk subgroup might adopt to immunotherapy. Besides, it has been reported that IFNG, CD274, CD8, and Merck18 are positive biomarkers of immunotherapy, while T-cell exclusion signature and MDSC are negative biomarkers of immunotherapy [58,59]. It is noteworthy that upregulation of positive immunotherapy response biomarkers (IFNG, CD274, CD8, and Merck18) and downregulation of negative biomarkers (T-cell exclusion signature and MDSC) in the high-risk subgroup provided an essential grounding for immunotherapy response prediction.

In addition, tumor type, age, cancer status, grade, and risk score were demonstrated to be capable of acting as independent prognostic indicators. Then, a nomogram plot involving the above independent prognostic indicators was plotted for quantitatively predicting the survival probability of LGG patients. As for the validation of the nomogram, the AUC values of the ROC curves were satisfied, which suggests a great prognostic ability of the nomogram.

The present study has several limitations. First, the FPRG-based signature was created and verified based on retrospective data from TCGA and CGGA databases. Further large-scale prospective clinical studies are required to evaluate its effectiveness and practicability. Besides, more well-designed basic research experiments are warranted to highlight the crucial role of ferroptosis and pyroptosis in the occurrence and development of LGG.

## CONCLUSION

The present research suggests that the 6-FPRG-related signature may serve as an effective indicator to anticipate the prognosis and immunotherapy response of LGG patients.

## SUPPLEMENTAL DATA

Supplementary tables can be found on this link: https://www.bjbms.org/ojs/index.php/bjbms/article/view/6888/2468

**Supplementary Table S1: Raw Data of FPRGs in TCGA Cohort**

**Supplementary Table S2: Raw Data Of FPRGs in CGGA COHORT**

**Supplementary Table S3: Immune Checkpoint Gene List**

**Supplementary Table S4: The Expression Levels of the 6 FPRGs and Riskscore Calculation In TCGA Cohort**

**Supplementary Table S5: The Expression Levels of the 6 FPRGs and Riskscore Calculation In CGGA Cohort**
